# Phytic Acid and Mineral Biofortification Strategies: From Plant Science to Breeding and Biotechnological Approaches

**DOI:** 10.3390/plants9050553

**Published:** 2020-04-26

**Authors:** Eleonora Cominelli, Roberto Pilu, Francesca Sparvoli

**Affiliations:** 1 Institute of Agricultural Biology and Biotechnology, Consiglio Nazionale delle Ricerche, Via E. Bassini 15, 20133 Milan, Italy; 2 Department of Agricultural and Environmental Sciences—Production Landscape, Agroenergy, Università degli Studi di Milano, Via G. Celoria 2, 20133 Milan, Italy

**Keywords:** biofortification, *low phytic acid* (*lpa*) mutants, metal transporter, mineral deficiencies, phytic acid

## Abstract

Mineral deficiencies, particularly for iron and zinc, affect over two billion people worldwide, mainly in developing countries where diets are based on the consumption of staple crops. Mineral biofortification includes different approaches aimed to increase mineral concentration and to improve mineral bioavailability in the edible parts of plants, particularly the seeds. A multidisciplinary approach, including agronomic, genetic, physiological, and molecular expertise, is necessary to obtain detailed knowledge of the complex homeostatic mechanisms that tightly regulate seed mineral concentrations and the molecules and mechanisms that determine mineral bioavailability, necessary to reach the biofortification objectives. To increase bioavailability, one strategy is to decrease seed content of phytic acid, a highly electronegative molecule present in the cell that chelates positively charged metal ions, many of which are important for human nutrition. All the contributions of the current Special Issue aim at describing new results, reviewing the literature, and also commenting on some of the economic and sociological aspects concerning biofortification research. A number of contributions are related to the study of mineral transport, seed accumulation, and approaches to increase seed micronutrient concentration. The remaining ones are mainly focused on the study of *low phytic acid* mutants.

## 1. Introduction

In 2015, we edited a *Plants* Special Issue on “Phytic Acid Pathway and Breeding in Plants”, with the aim to provide a unique compendium that highlighted new developments in our understanding of how perturbation in phytic acid (PA) synthesis and accumulation contributes to plant function, growth, and response to the environment. After 4 years, we believe that the development of biofortified crops to respond to mineral deficiencies is still challenging. For this reason, we decided to launch a new Special Issue on “Phytic Acid and Mineral Biofortification Strategies: From Plant Science to Breeding and Biotechnological Approaches” in which we collected articles and reviews providing new knowledge and technical advances in the field. 

The review from Jha and Warkentin [1] can be considered as an introduction to the topics of the present Special Issue. Although it focuses on the approaches used to biofortify pulses for different key micronutrients, it also provides a general overview of the different strategies to tackle micronutrient deficiency. The authors first describe the requirements and functions of the different key micronutrients in humans and the negative impacts of their deficiency in the diet. Then, different approaches that can be used to improve micronutrient content and absorption from the diet are presented. These approaches include dietary diversification, the use of food supplements, food fortification, and biofortification. 

The main objectives for crop seed biofortification are: (i) to increase seed micronutrient concentration; (ii) to decrease seed content of antinutritional factors that reduce micronutrient bioavailability, mainly PA.

To reach the first objective, it is important to acquire basic knowledge of the genes involved in mineral transport, such as the *Vacuolar Iron Transporter-Like* (*VTL*) genes described in the article from Sharma et al. [2], and of how minerals accumulate into seeds, the focus of the article by Pongrac et al. [3]. An overview of breeding approaches used to develop pulses with increased seed mineral concentration is presented in the already cited review [1]. 

The other contributions of the present Special Issue cover different aspects related to the second objective of crop biofortification. A very fascinating review from Raboy evaluates the possible economic and social impact of *low phytic acid* (*lpa*) mutants [4]. A review and a commentary are focused on *lpa* mutants in transporters [5,6]. Four articles describe the isolation and characterization in different crops of *lpa* mutants [7,8,9,10], derived from mutagenized populations [7,9,10] or from the genome editing technology approach [8]. An overview of the role of inositol pyrophosphates and useful recommendations for the development of novel *lpa* mutants is presented by Freed et al. [11]. 

## 2. Mineral Transport, Seed Accumulation, and Breeding to Increase Concentration in Seeds

### 2.1. Mineral Transport and Seed Accumulation

Seed iron is mainly stored in vacuoles. Hence, improving iron uptake into the vacuole is a valuable alternative strategy to increase total iron content. For this reason, the role of vacuolar iron transporters needs to be addressed and exploited. The article by Sharma et al. describes the isolation and preliminary characterization of the family of the *VTL* genes in hexaploid wheat. The authors report data on phylogenetic analysis and on a quantitative expression analysis of *VTL* genes in response to iron surplus and deficiency, under zinc, manganese, and copper deficiency, and under heavy metals treatments [2]. Particularly, 23 wheat *VTL* gene sequences were identified that can be phylogenetically distinguished from the *Vacuolar Iron Transporters* (*VIT*) ones and are grouped as 4 *VTL* genes due to the occurrence of homeologs. The expression data in response to treatments with different concentrations of minerals suggest that these genes have an important role in mineral homeostasis [2].

The knowledge of mechanisms involved in mineral accumulation, in terms of tissue-specificity, speciations, and ligand compounds, is very important to set up a precise biofortification program. In the wheat ear, awns (bristle-like structures extending from lemmas) have transpiration and photosynthetic activity. Hence, their presence could contribute to the translocation of elements taken up by roots on the one hand and/or to the phloem-driven (re)allocation of assimilates on the other hand, thereby affecting mineral element density in the grain. The study by Pongrac et al. presents a comparison of mineral element composition between awned and awnletted (those that have short or no awns) wheat cultivars. Moreover, tissue-specific iron speciation and iron ligands in the cultivars contrasting for seed iron content were also investigated using micro X-ray absorption near edge structure (micro-XANES) [3]. The authors found that among the 20 different cultivars, the awnletted ones showed lower whole-grain concentrations of calcium and manganese, but higher iron concentration, compared to the awned cultivars. Interestingly, no differences were observed either in iron speciation (the percentages of ferric and ferrous iron are similar in the four most contrasting analyzed awned and awnletted cultivars) or in terms of ligands, as on average 53% of the iron is in a phytate form. On the contrary, there was a distinct tissue-specificity in iron speciation and ligands, with the pericarp containing the largest proportion of ferric species with only non-phytate ligands, as also in the nucellar projection. In other tissues, such as the aleurone, scutellum, and embryo, iron was predominantly bound to phytate. The authors conclude that, as iron bioavailability is dependent on iron ligands, its bioavailability in wheat is tissue specific. Further investigation on the genetic and/or metabolic reasons behind the observed differences is needed [3].

### 2.2. Germplasm Screening: Genetic Variation and Identification of Genomic Regions and Molecular Markers (Quantitative Trait Loci (QTLs), Single Nucleotide Polymorphisms (SNPs))

The increase of seed micronutrient concentration can be achieved through agronomic interventions, genetic engineering, and plant breeding. The first two approaches are briefly summarized in the review by Jha and Warkentin [1]. However, a long section of the review is dedicated to conventional plant breeding approaches that have been used to biofortify pulses, particularly common bean, lentil, chickpea, mungbean, and pea, for some minerals (iron, zinc, and selenium), carotenoids, and folates. The advantages of using conventional plant breeding, compared to the genetic engineering strategy, are the relatively low costs and the high acceptability by consumers. The first step in this kind of approach is the screening for genetic variability for micronutrients’ seed concentration. The authors report that a significant effect of the genotype in the determination of micronutrient seed concentration has been shown for the different micronutrients in the various pulses, although the environment (weather and soil factors, such as aeration, water availability, pH, and texture) may have significant effects, for example, in the case of zinc and selenium seed concentrations. Moreover, for the pulses studied, they report the identification of genomic regions (Quantitative Trait Loci, QTLs) and molecular markers, mainly Single Nucleotide Polymorphisms (SNPs), which can be used in marker-assisted selection procedures, aimed at improving micronutrient seed concentration. In some cases, candidate genes involved in the accumulation of the different micronutrients are described. The authors mention the different crops, not only pulses, with increased micronutrient concentration that have been released in recent years in developing countries, mainly thanks to the activity of HarvestPlus, an initiative of the Consultative Group on International Agricultural Research (CGIAR), started in 2003 to enrich various major crops with iron, zinc, and vitamin A. Evidence that the introduction of some of these crops in the diet has helped in overcoming nutrient deficiency is well documented [1].

## 3. Decreasing Antinutritional Compounds Concentration: Four Decades of Research and Novel Perspectives for *lpa* Mutants 

### 3.1. Some Possibilities to Redeem the so far Neglected lpa Crops?

PA is the most abundant form of phosphorus (P) occurring in seeds. However, it is a “non-available” form of P for monogastric animals devoid of phytase (poultry, swine, fish). Moreover, it is a strong cation chelator, reducing the bioavailability of cations important for nutrition. PA is also a very important signaling molecule involved in different regulatory processes during plant development and responses to different stimuli [12]. Different contributions to the present Special Issue treat aspects related to PA and *lpa* mutants [4,5,6,7,8,9,10].

Different *lpa* mutants have been isolated and characterized so far in different species, starting from the first ones, the maize *lpa1-1* and *lpa2-1*, isolated in the early 1990s in the USDA-ARS laboratory of Raboy, but none of them has been commercialized, as the scientist underlines, with a certain regret, in his very interesting commentary [4]. The commentary is strongly felt, as the author is “a person intimately involved in the entire process across a 40-year period,” as written in his/her report by one of the anonymous reviewers of the manuscript (the review reports are publicly available at the webpage of the commentary). As still observed by one of the two reviewers, the view presented in this commentary is multidisciplinary, as scientific, economic, and social aspects of the subject are discussed, “encompassing the issues faced by all breeders (and probably agronomists as well) attempting to develop new materials with significant social benefit but difficult to capture short term economic benefit when favored alternatives exist with the reverse tendencies”. The author underlines that these mutants have some potential advantages, mainly (i) improving phosphorus management in non-ruminant production, contributing to enhance sustainability and reduce animal waste P, and (ii) increasing mineral bioavailability as a strategy to combat mineral deficiencies, as shown by different studies. Nevertheless, these mutants have received very little interest. The author thinks that the reasons for this are primarily due to the reduced yield (5–10% decrease) and field performance that characterize some of these mutants, together with the criticism that reducing PA is not wholly advantageous as it may also have positive nutritional benefits (antioxidant and anticancer properties, shown through *in vitro* studies [13], although it has been shown that no phytate is present in human biofluids [14]) that might be lost in the *lpa* mutants. Moreover, another simple explanation may be the tendency to use a conservative approach to crop improvement strategies for crops that provide staple foods to at-risk populations in developing countries. However, no support or time to improve the agronomic performance of these mutants has been provided. Some alternatives that have been preferred in recent years exist. Concerning methods to increase P for feed, it can be directly added or phytase can be used to increase the component of available P. The author says that these methods have been preferred to the use of *lpa* crops, without calculating the possible long-term money-saving deriving from using the *lpa* crops. Moreover, the positive results from animal nutrition studies when animals are fed *lpa* crops (for example: leaner pigs, with enhanced muscle density and less backfat when fed *lpa* maize; eggs with reduced cholesterol from hens fed *lpa* maize) ironically pushed farmers to apply phytase superdoses, also in this case not considering the long-term money-saving if, instead, *lpa* crops might have been used [4]. These savings would have overcome the reduced yield problem with the further advantages of having more nutritious crops. Raboy is also quite critical of the HarvestPlus program and the international agricultural centers participating in the Gates Foundation that concentrated all their efforts only to promote biofortification through breeding crops for elevated micronutrient density and, therefore, pushed the development and promotion of *lpa* crops to the sidelines, although a combination of both approaches would likely give the most promising results. 

After the publication of this Special Issue, an article has been published by different authors, including scientists from HarvestPlus and CIAT (a CGIAR institute), comparing the retention of iron and zinc when preparing common household recipes with conventional, biofortified, or *lpa* common beans [15]. The retention of iron was very high and similar using the different bean genotypes, while *lpa* beans exhibited lower retention for zinc. Further studies are needed to understand this difference. However, the authors encourage the development of beans with an increased mineral content combined with a low PA trait, and also with low concentrations of specific polyphenolic compounds, as the research target for the next generation of biofortified beans [15]. This publication can lay the foundation for a brighter future for *lpa* crops.

### 3.2. lpa Mutants in Different Classes of Transporters: Not Always so Obvious

PA reduction can be achieved with mutations in different types of transporters that control PA transport to the vacuole (MRP-type ABC transporters), or by modifying inorganic P (P_i_) availability for PA synthesis through mutations in transporters involved in P_i_ loading and organ/intracellular distribution (SULFATE TRANSPORTER 3;3 -SULTR3;3- and SULTR3;4, members of the group 3 of putative sulfate transporters) or by P_i_ acquisition and mobilization during seed development (PHOSPHATE TRANSPORTER 1;4, PHT1;4). The review by Cominelli et al. is focused on the description of genes, proteins, and mutants of these different transporters in cereals and legumes [5]. Particular attention is dedicated to those mutants devoid of negative pleiotropic effects, such as mutants affected by the common bean *MRP1* gene and by the rice and barley *SULTR3;3* and rice *SULTR3;4* genes, suggesting strategies to develop useful *lpa* mutants in other species as well [5]. 

Sacchi and Nocito in their opinion paper propose a deeper discussion on group 3 putative sulfate transporters and suggest some hypotheses to unveil the links between sulfate and P accumulation in seeds [6]. The fact that genes predicted to encode for sulfate transporters, when mutated, cause a *lpa* phenotype is anything but obvious. Differently from other sulfate transporters, for which capability to transport sulfate has mainly been proven through complementation of yeast mutants, the function of SULTR3s has only been hypothesized based on their sequence homologies. The rice and Arabidopsis SULTR3;4 proteins are able to transport phosphate and not sulfate, as recently shown [16,17], explaining the *lpa* phenotype of the rice mutant. However, the OsSULTR3;3 protein does not show either phosphate or sulfate transport activity. Some hypotheses on the link between sulfate and phosphate homeostasis and the development of the *lpa* phenotype in *sultr3;3* mutants are proposed in the opinion paper [6].

### 3.3. Response to P Fertilization

Seed PA content is affected by the amount of supplied P in various crops [18,19,20]. In this Issue, two articles investigate the effects of P fertilization in genotypes differing in their PA content, particularly in a soybean *lpa* mutant compared with two normal-phytate cultivars [10] and in two rice cultivars described for their contrasting grain PA content [9]. 

In the first article, Taliman et al. [10] compare different parameters, such as dry weight, photosynthetic rate, dinitrogen fixation, mineral accumulation, and grain yield between a soybean *lpa* line and two varieties normally cultivated in Japan (wild type, wt) in response to high and low P fertilization. The authors generally observed increased plant performance and yield at higher P concentration in all three genotypes but very little difference between *lpa* and wt genotypes. Seed yield was higher in the *lpa* line than in the normal-phytate cultivars at both fertilization doses. The results show that the already positive properties of *lpa* seeds in terms of increased mineral cations bioavailability can be also accompanied by good agronomic performance [10].

In the second article, Fukushima et al. [9] analyzed the response to P fertilization of two previously selected rice genotypes differing for their PA content with the World Rice Core Collection 5 (WRC 5) genotype showing the lowest and WRC 6 showing the highest PA content among different accessions [21]. The authors reported that differences in PA content between the two contrasting cultivars were observed only under standard P fertilization conditions, while, if two different doses of P fertilizer were applied at different developmental stages (at seedling or heading stage) an increase in PA content was observed in both genotypes, highly reducing the differences between the two genotypes. Interestingly, the expression level of the *myo-inositol 3-phosphate synthase 1* (*INO1*) gene was suggested to be the genetic basis explaining the natural variation in PA accumulation in rice. Although the DNA sequences of the coding region and a putative promoter region of 1000 bp of the *INO1* gene were identical between WRC 5 and WRC 6, the gene is more expressed in the WRC 6 accession than in the WRC 5 one. Moreover, the *INO1* gene transcript accumulation increased in response to P fertilizer only in the WRC 6 accession. The authors hypothesized the existence of different regulatory mechanisms of PA content besides the DNA mutation in the *INO1* gene [9].

### 3.4. lpa Mutants: Isolation and Characterization of New Mutants and Description of a Novel Screening Method in Maize

The articles by Jiang et al. and Borlini et al. focus on the isolation and characterization of novel *lpa* mutants in rice and maize, respectively; in the second article, a particularly easy new screening method for maize *lpa* mutants is also described [7,8]. 

Most of the *lpa* mutants have been isolated by screening mutagenized populations and few examples using transgenic approaches have been also reported [12]. Some maize *lpa* mutant lines were obtained through a genome-editing based method, when this technology was not so popular as today [22] and very recently barley mutant lines have been isolated through the same technology [23]. In the article presented by Jiang et al. [8], the CRISPR/CAS9 method was used to generate four rice mutants in the *OsITPK6* gene, coding for inositol 1,3,4-triphosphate 5/6 kinase. Very recently barley *lpa* allelic variants have been isolated through the same technology. Jiang et al. described that the decrease in PA content and the severity of the negative pleiotropic effects depended on the induced mutations, with the three frameshift mutations resulting in a major reduction in PA content and in a stronger impact on plant germination, growth, reproduction, and abiotic stress tolerance compared to the effects due to the 6-bp in-frame mutation. There is a discrepancy between results obtained from the present study and from a previous one [24], where another mutant affected in the same gene showed a higher decrease in PA content than reported for the mutant in the article by Jiang et al., but normal plant growth. Further studies on other *ositpk6* mutants could clarify this discrepancy and the role of this gene in plant growth and reproduction in addition to its role in PA biosynthesis [8]. 

Different papers have reported the isolation of *lpa* mutants in different species by the screening of F_2_ mutagenized populations through the disruption of the seeds analyzed by Chen’s assay [12]. In the paper by Borlini et al. [7], it was proposed to directly identify the putative mutant seeds by a cheap and fast screening method based on the lower density of *lpa1* seeds with respect to the wild type, as reported in previous papers, where among the pleiotropic effects associated with the *lpa* mutation it was also shown that there was a reduction of seed density in maize and rice. This assay was able to identify the *lpa* mutant seeds because the *lpa1* seeds can float in a concentrated sugar solution (density 1.218–1.222 g/cm^3^) due to their lower density, unlike the wild sibs that sink [7]. Hence, this method could be used in massive screening of mutagenized populations with the aim to isolate allelic variants at the *lpa* locus.

### 3.5. Inositol Pyrophosphate: Suggested Strategies for the Development of Novel lpa Mutants

In the cell, a small pool of PA can be further phosphorylated to form inositol pyrophosphates (PP-InsP), containing one or two diphosphate groups (InsP_7_ and InsP_8_), through the activity of inositol triphosphate kinase (ITPK) enzymes that phosphorylate PA to InsP_7_ and the diphosphoinositol-pentakisphosphate kinases (PPIP5Ks) that phosphorylate InsP_7_ to InsP_8_. PP-InsP have important roles in energy metabolism, hormone signaling (mainly jasmonate), and P_i_ sensing. It has been shown that different Arabidopsis *lpa* mutations, affecting PA biosynthetic genes, also cause a reduction in the content of InsP_8_ and in some cases of InsP_7._ Starting from this point, Freed et al. recommend the breeders aiming at developing *lpa* mutants to take into account this aspect to avoid negative pleiotropic effects that may reduce pathogen defense, mediated by jasmonate, and affect phosphate homeostasis [11]. To overcome this possibility, one strategy is to develop transgenic *lpa* lines using tissue-specific promoters active only in the seed. However, also in the seed, InsP_7_ and InsP_8_ may have important roles in phosphate homeostasis not yet investigated. On the other hand, the Arabidopsis *mrp5* mutant, affecting the PA-MRP vacuolar transporter, shows increased content of both InsP_7_ and InsP_8_, representing an interesting target for the development of new *lpa* mutants not compromised in P_i_ homeostasis and in jasmonate signaling. In this way, the review intends to bridge the gap between the basic science aspects of PP-InsP synthesis and function and the breeding/engineering strategies aimed at developing *lpa* crops [11].

## 4. Conclusions

Although micronutrient malnutrition is still a challenging problem, progress has been achieved in the development of biofortified crops, either by enhancing the content of key microelements such as iron and zinc or by developing *lpa* mutants with good agronomic performance [1]. Enhancing the content of key microelements has been achieved essentially by breeding, while studies on the elucidation of the mechanisms, and therefore of the genes, involved in micronutrient uptake and efficient storage in the seed are still in progress [1,2,3].

Advances have been achieved in understanding the function of a number of structural genes involved in PA biosynthesis [7,8,11] and new genes, not obviously correlated to PA biosynthesis and storage, have been discovered to play a role in PA accumulation in the seed [5,6]. Furthermore, there is increasing evidence that many negative pleiotropic effects commonly associated with *lpa* mutants may be overcome by efficient breeding, thus making reasonable and convenient the production of *lpa* mutants [4,9,10]. To reinforce this convenience, there are also social and economic considerations, as clearly explained in the review by Raboy [4]. 

In conclusion, it is quite clear that, at the moment, the most promising strategy to produce effective biofortified crops is by combining seed PA reduction (*lpa* mutants) with increased seed mineral content, as a number of results provide evidence showing that PA is still the main limiting factor to cations’ bioavailability in the diet of humans and monogastric animals [15].
